# Hypoimmune induced pluripotent stem cell–derived cell therapeutics treat cardiovascular and pulmonary diseases in immunocompetent allogeneic mice

**DOI:** 10.1073/pnas.2022091118

**Published:** 2021-07-09

**Authors:** Tobias Deuse, Grigol Tediashvili, Xiaomeng Hu, Alessia Gravina, Annika Tamenang, Dong Wang, Andrew Connolly, Christian Mueller, Beñat Mallavia, Mark R. Looney, Malik Alawi, Lewis L. Lanier, Sonja Schrepfer

**Affiliations:** ^a^Division of Cardiothoracic Surgery, Department of Surgery, Transplant and Stem Cell Immunobiology Laboratory, University of California, San Francisco, CA 94143;; ^b^Department of Cardiovascular Surgery, University Heart Center Hamburg, 20246 Hamburg, Germany;; ^c^German Center for Cardiovascular Research (DZHK) partner site Hamburg/Kiel/Luebeck, 20246 Hamburg, Germany;; ^d^Sana Biotechnology Inc., South San Francisco, CA 94080;; ^e^Department of Pathology, University of California, San Francisco, CA 94143;; ^f^Horae Gene Therapy Center, University of Massachusetts, Worcester, MA 01605;; ^g^Department of Pediatrics, University of Massachusetts, Worcester, MA 01605;; ^h^Department of Medicine, University of California, San Francisco, CA 94143;; ^i^Department of Laboratory Medicine, University of California, San Francisco, CA 94143;; ^j^Bioinformatics Core, University Medical Center Hamburg-Eppendorf, 20246 Hamburg, Germany;; ^k^Department of Microbiology and Immunology and the Parker Institute for Cancer Immunotherapy, University of California, San Francisco, CA 94143

**Keywords:** hypoimmune stem cells, immune evasion, cell therapy

## Abstract

The emerging field of regenerative cell therapy is still limited by the few cell types that can reliably be differentiated from pluripotent stem cells and by the immune hurdle of commercially scalable allogeneic cell therapeutics. Here, we show that gene-edited, immune-evasive cell grafts can survive and successfully treat diseases in immunocompetent, fully allogeneic recipients. Transplanted endothelial cells improved perfusion and increased the likelihood of limb preservation in mice with critical limb ischemia. Endothelial cell grafts transduced to express a transgene for alpha1-antitrypsin (A1AT) successfully restored physiologic A1AT serum levels in mice with genetic A1AT deficiency. This cell therapy prevented both structural and functional changes of emphysematous lung disease. A mixture of endothelial cells and cardiomyocytes was injected into infarcted mouse hearts, and both cell types orthotopically engrafted in the ischemic areas. Cell therapy led to an improvement in invasive hemodynamic heart failure parameters. Our study supports the development of hypoimmune, universal regenerative cell therapeutics for cost-effective treatments of major diseases.

## Significance

Precise gene editing allows engineering of immune receptors and ligands to reduce the immunogenicity of cells, and strategies for the generation of immune-evasive stem cell sources are currently being developed. This article describes the translational aspect of generating universally transplantable, disease-specific, therapeutic cell products. We provide proof of concept that immune-engineered cells can treat major cardiovascular and pulmonary diseases in fully allogeneic subjects without utilizing any immunosuppression. The translational aspect is emphasized by showing improvements in clinically relevant outcome measures, which are widely used in human trials. Depending on the feasibility of large-scale manufacturing of universal cell therapeutics, this approach could enable cost-effective cell therapy.

Affordability will promote the development and commercialization of cell therapy products, and optimization of cost of goods should be addressed from the very beginning to facilitate broader adoption for patient treatment (1). From a population health standpoint, only allogeneic products will allow manufacturing at large scale and with rigorous quality standards necessary for the treatment of large patient populations (2). However, the need for immunosuppression is a major long-term concern and only acceptable for early proof-of-concept clinical studies. The emergence of immune engineering now offers a unique opportunity to develop universal off-the-shelf products for all patients and all tissue types. We (3) and others (4) have developed immune engineering concepts that allow cells to evade rejection in immunocompetent allogeneic recipients. We now present applications of this technology and report successful treatments of different diseases with hypoimmune (HIP) induced pluripotent stem cell (iPSC)–derived cells in allogeneic mice without the need for any immunosuppression.

## Results

### Generation of Universal, Hypoimmune Endothelial Cells and Cardiomyocytes.

**Fig. 1.**
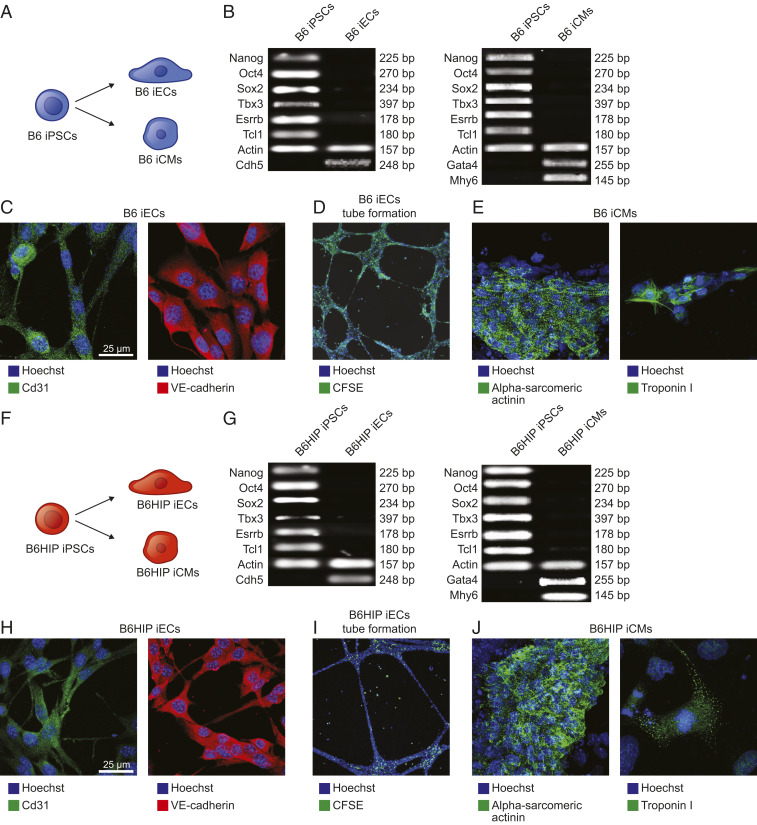
Differentiation of B6 and B6HIP iPSCs into iCMs and iECs. (*A*) B6 iPSCs were differentiated into iECs and iCMs. (*B*) During the differentiation, the cells lost their typical pluripotent features of iPSCs and B6 iECs adopted genetic markers of endothelial cells and B6 iCMs of cardiomyocytes (representative gels of two independent experiments). (*C* and *D*) B6 iECs expressed Cd31 and VE-cadherin (*C*, representative pictures of five independent experiments) and formed tubular structures in vitro (*D*, representative pictures of five independent experiments). (*E*) B6 iCMs expressed alpha-sarcomeric actinin and troponin I (representative pictures of five independent experiments). (*F*) B6HIP iPSCs were differentiated into iECs and iCMs. (*G*) During the differentiation, the cells lost their typical pluripotent features of iPSCs and B6HIP iECs adopted genetic markers of endothelial cells and B6HIP iCMs of cardiomyocytes (representative gels of two independent experiments). (*H* and *I*) B6HIP iECs expressed Cd31 and VE-cadherin (*H*, representative pictures of five independent experiments) and formed tubular structures in vitro (*I*, representative pictures of five independent experiments). (*J*) B6HIP iCMs expressed alpha-sarcomeric actinin and troponin I (representative pictures of five independent experiments).

To generate hypoimmune, universally compatible mouse cell products, we utilized a recently developed immune-editing strategy. Wild-type (WT) C57BL/6 (B6) mouse iPSCs underwent a three-step gene editing protocol (3). The *B2m* and *Ciita* genes were targeted for disruption with Cas9 nuclease and guides to deplete major histocompatibility complex (MHC) class I and class II expression and Cd47 was overexpressed using lentiviral transduction. These B6HIP iPSCs as well as their parental B6 iPSCs were transduced to express firefly luciferase (FLuc) for subsequent bioluminescence imaging (BLI) studies to quantitatively assess their survival after transplantation. Both B6 and B6HIP iPSCs were differentiated into iPSC-derived endothelial cells (iECs) and cardiomyocytes (iCMs) (Fig. 1). The purity of the generated derivatives was >80% for iECs and >95% for iCMs and similar between B6 and B6HIP (*SI Appendix*, Fig. S1 *A–D*).

### HIP iECs for the Preservation of Critically Ischemic Hindlimbs.

**Fig. 2.**
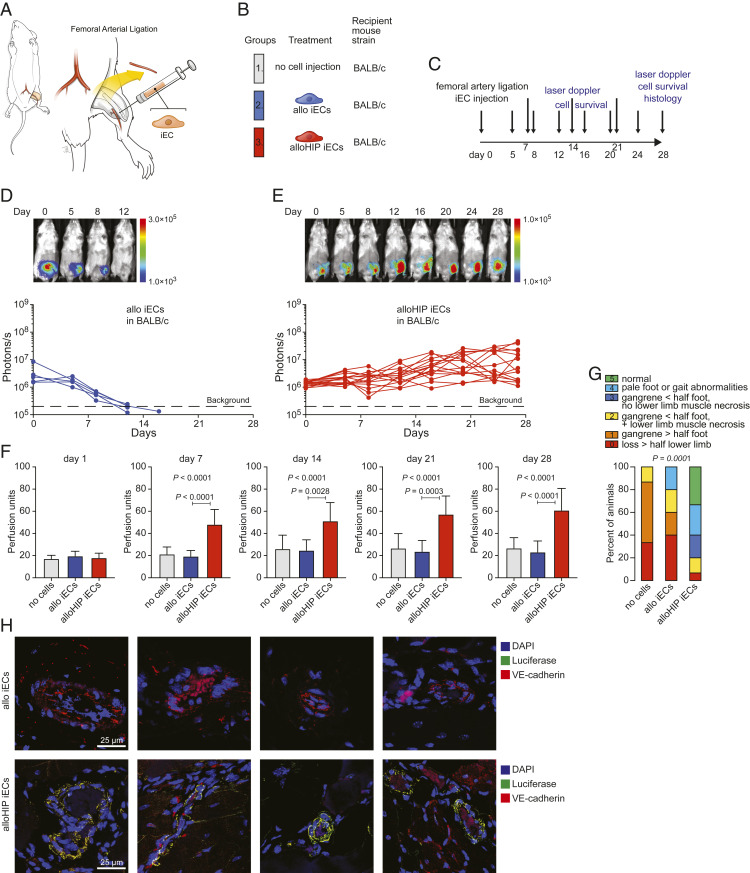
Allogeneic HIP iECs facilitate ischemic limb preservation. (*A*) In BALB/c mice, the superficial femoral artery was ligated and partially resected to induce left lower limb ischemia. (*B*) Mice were left untreated or received fan-shaped injections of allo or alloHIP iECs into the surrounding tissue. (*C*) The study protocol included assessment of iEC survival and laser Doppler perfusion imaging and histology after 28 d. (*D* and *E*) The survival of FLuc^+^ iEC grafts was longitudinally followed by BLI. All allo iEC grafts were rejected over 15 d (*D*, 5 animals), while all alloHIP iEC grafts survived and some grafts even showed proliferation (*E*, 15 animals). BLI signals of individual animals are plotted, representative pictures are shown. (*F*) Left lower extremity perfusion was serially assessed by laser Doppler imaging and showed an improvement over time only after transplantation of alloHIP iECs (mean ± SD, 15 animals with no cell injection, 5 animals in the allo group, and 15 animals in the alloHIP group; ANOVA with Bonferroni post hoc test). (*G*) The sequelae of CLI after 28 d were graded according to a standardized scoring system and showed improved limb preservation with alloHIP iEC treatment (parts of whole graphs, 15 animals with no cell injection, 5 animals in the WT group, 15 animals in the HIP group; Kruskal–Wallis test). (*H*) Immunofluorescence staining showed no engrafted FLuc^+^ allo iECs in allogeneic BALB/c recipients, but engraftment of FLuc^+^ alloHIP iECs located along the endothelial layer of larger and smaller intramuscular vessels. Costaining showed that transplanted cells retained their VE-cadherin expression (representative pictures of five independent experiments).

First, the efficacy of transplanted B6HIP iECs to salvage hindlimbs in allogeneic BALB/c mice with critical limb ischemia (CLI) was evaluated. Previous studies with endothelial progenitor cells in CLI have used autologous settings (5) or immunocompromised recipients (6, 7). BALB/c mice (H-2^d^), which are fully MHC mismatched to the B6 (H-2^b^) iECs, underwent ligation and excision of their left proximal superficial femoral artery (8) (Fig. 2*A*). The animals were left untreated or received fan-shaped injections of allogeneic B6 (allo) or allogeneic B6HIP (alloHIP) iECs and followed for 28 d (Fig. 2 *B* and *C*). Cell survival was assessed with BLI. All allo iEC grafts were rejected within 15 d (Fig. 2*D*), whereas all alloHIP iEC grafts survived and showed some proliferation with increasing BLI signals (Fig. 2*E*). Doppler imaging showed vastly reduced perfusion of the ischemic legs on the first day after the procedure in all groups (Fig. 2*F* and *SI Appendix*, Fig. S2). The perfusion index only increased in animals receiving alloHIP iECs and persistently showed better perfusion than in animals receiving allo iECs throughout the study period. The severity of ischemic sequelae was assessed using a standardized mouse limb ischemia grading scale (9). All animals receiving no cell therapy and the vast majority of animals receiving allo iEC grafts showed gangrenous lesions of different extents. Animals in the alloHIP iEC group, in contrast, showed less severe lesions, more than half of which were nongangrenous (Fig. 2*G* and *SI Appendix*, Fig. S3). The thighs were recovered and the area surrounding the excised femoral artery in which the cells were injected was sectioned (*SI Appendix*, Fig. S4). We found fatty replacement of muscle with endomysial fat cells and occasional dystrophic calcifications. In alloHIP animals, we saw spindle cells of endothelial morphology, some outgrowing from Matrigel remnants. Some also showed small clusters of poorly differentiated cells. Using immunofluorescence imaging, we were able to detect FLuc^+^ graft cells in many vascular structures, particularly small and larger arteries, in all alloHIP iEC recipients (Fig. 2*H*). The incorporated graft cells were VE-cadherin^+^ and located along the endothelial cell layer in these vessels. We did not find any FLuc^+^ allo iECs in any of the animals. These data show that alloHIP iECs survive and proliferate in allogeneic animals, incorporate into vascular structures, improve limb perfusion, and reduce the incidence of gangrenous limb complications.

### HIP iECs for the Treatment of A1AT.

**Fig. 3.**
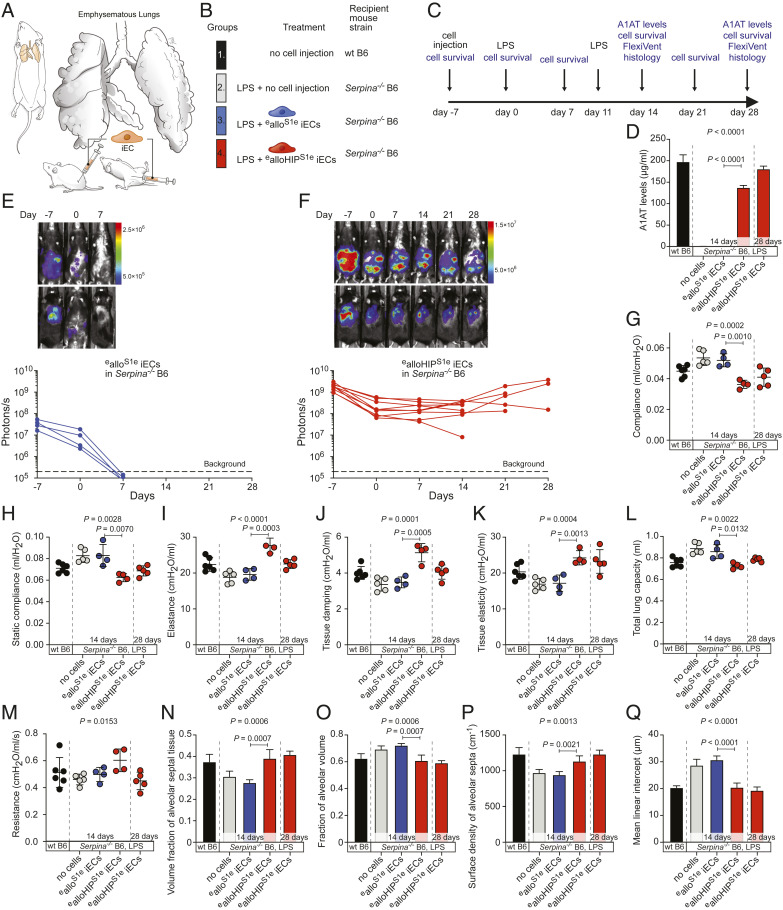
A1AT-releasing allogeneic HIP iECs prevent emphysema development in Serpina^−/−^ B6 mice. (*A*) *Serpina*^*−/−*^ B6 mice were challenged with LPS via their airways to trigger emphysematous lung disease. (*B*) Some *Serpina*^*−/−*^ B6 mice were treated with MHC-engineered A1AT-secreting allogeneic (^e^allo^S1e^) or allogeneic HIP (^e^alloHIP^S1e^) iECs 7 d before the LPS challenge. Healthy WT B6 animals served as controls. (*C*) The study protocol included monitoring of graft survival, as well as functional and morphologic lung assessments. (*D*) Serum A1AT levels were quantified in all groups (mean ± SD, six animals in WT B6, five animals in *Serpina*^*−/−*^ B6 LPS without cell injections at day 14, four animals in *Serpina*^*−/−*^ B6 LPS ^e^allo^S1e^ iECs at day 14, four animals in *Serpina*^*−/−*^ B6 LPS ^e^alloHIP^S1e^ iECs at day 14, and five animals in *Serpina*^*−/−*^ B6 LPS ^e^alloHIP^S1e^ iECs at day 28; ANOVA with Bonferroni post hoc test for 14-d groups). (*E* and *F*) The survival of FLuc^+ e^allo^S1e^ iECs (*E*, four animals) and ^e^alloHIP^S1e^ iECs (*F*, nine animals) in *Serpina*^*−/−*^ B6 recipients was longitudinally followed by BLI. BLI signals of individual animals are plotted, representative pictures are shown. (*G*–*M*) FlexiVent lung physiology assessments were done after 14 and 28 d (scatter dot plots, mean ± SD, six animals in WT B6, five animals in *Serpina*^*−/−*^ B6 LPS without cell injections at day 14, four animals in *Serpina*^*−/−*^ B6 LPS ^e^allo^S1e^ iECs at day 14, four animals in *Serpina*^*−/−*^ B6 LPS ^e^alloHIP^S1e^ iECs at day 14, and five animals in *Serpina*^*−/−*^ B6 LPS ^e^alloHIP^S1e^ iECs at day 28; ANOVA with Bonferroni post hoc test for 14-d groups). (*N*–*Q*) Stereological lung assessments were done in all groups (mean ± SD, six animals in WT B6, five animals in *Serpina*^*−/−*^ B6 LPS without cell injections at day 14, four animals in *Serpina*^*−/−*^ B6 LPS ^e^allo^S1e^ iECs at day 14, four animals in *Serpina*^*−/−*^ B6 LPS ^e^alloHIP^S1e^ iECs at day 14, and five animals in *Serpina*^*−/−*^ B6 LPS ^e^alloHIP^S1e^ iECs at day 28; ANOVA with Bonferroni post hoc test for 14-d groups).

Next, we aimed to use iEC grafts as factories to replace a crucial missing factor responsible for disease. We chose a mouse model of A1AT deficiency that quickly develops both structural and functional changes of lung disease (10). In B6 mice with quintuple *Serpina1a–e* knockout, a mild lipopolysaccharide (LPS) challenge that is well tolerated in WT B6 mice, leads to emphysema development due to the unabated activity of the secreted neutrophil elastase (NE) from recruited polymorphonuclear cells. To turn iECs into enzyme factories, both B6 and B6HIP iECs were engineered to secrete mouse A1AT. The mouse *Serpina1e* (S1e) cDNA sequence was synthesized and cloned into a lentiviral vector with zeocin resistance, B6 and B6HIP iECs were transduced, and antibiotic-selected pools of B6^S1e^ and B6HIP^S1e^ iECs were expanded. Since the *Serpina*^*−/−*^ mice were bred on a B6 background and thus syngeneic to our iECs, we had to further engineer alloantigens into the iECs to make them allogeneic. We used two additional lentiviral particles carrying transgenes for H-2K^d^, a major allele of the BALB/c H-2^d^ genotype, and the BALB/c-variant of *Co3*, an immunogenic minor antigen of the mitochondrial DNA (11). Such MHC-engineered ^e^allo^S1e^ and ^e^alloHIP^S1e^ iECs showed the classic EC phenotype (*SI Appendix*, Fig. S5 *A* and *B*) and a similar purity (*SI Appendix*, Fig. S1 *E* and *F*) as the parental B6 and B6HIP iECs. A total of 1.5 × 10^5 e^allo^S1e^ and ^e^alloHIP^S1e^ iECs, as well as their parental and intermediary iEC populations, were plated for 24 h, and A1AT levels were measured (*SI Appendix*, Fig. S5 *C* and *D*). The ^e^allo^S1e^ and ^e^alloHIP^S1e^ iECs produced ∼50 ng A1AT in a day. With a half-life of A1AT in mice of approximately 4.5 d, we estimated that a cell number of 1.5 × 10^8^ A1AT-producing cells was necessary to restore and maintain physiologic A1AT serum levels, which are around 200 μg/mL. Seven days before induction of lung disease, *Serpina*^*−/−*^ B6 mice received 1.5 × 10^8 e^allo^S1e^ or ^e^alloHIP^S1e^ iECs in saline injected into the peritoneum and the subcutaneous tissue in the lumbar area to equally split the cell load (Fig. 3*A*). *Serpina*^*−/−*^ B6 mice without cell injection, as well as healthy WT B6 mice, served as controls (Fig. 3*B*). Lung disease was induced in all *Serpina*^*−/−*^ B6 groups with LPS instillation on day 0 (7 d after cell injection) and repeated on day 11 (Fig. 3*C*). After 14 d, the established time period to assess lung disease in LPS-treated *Serpina*^*−/−*^ B6 mice (10), all mice receiving the ^e^allo^S1e^ iECs and half of the animals receiving ^e^alloHIP^S1e^ iECs underwent FlexiVent evaluation. Five animals receiving ^e^alloHIP^S1e^ iECs were assessed on day 28 to have a consistent follow-up period with the other disease models in this study. Serum was drawn from animals after the FlexiVent. On day 14, A1AT was undetectable in both untreated *Serpina*^*−/−*^ mice and those receiving ^e^allo^S1e^ iECs. Cell therapy with ^e^alloHIP^S1e^ iECs, in contrast, was able to restore A1AT levels and 14-d and 28-d levels were well in the physiologic range (Fig. 3*D*). Cell survival was assessed by BLI. All recipients of ^e^allo^S1e^ iECs (Fig. 3*E*) rapidly rejected their allogeneic, MHC-engineered grafts within 7 d, whereas all ^e^alloHIP^S1e^ iECs showed survival throughout the observation period (Fig. 3*F*). Two animals of the 28-d group missed their last imaging due to technical difficulties. Pulmonary mechanics were assessed using a computer-controlled piston ventilator for standardized measurements. On day 14 of the study, untreated *Serpina*^*−/−*^ B6 mice showed ventilation patterns consistent with developing emphysema, including increased lung compliance and decreased elastance (Fig. 3 *G*–*M*). The coefficients of tissue damping and tissue elasticity were both reduced and the total lung capacity was increased. As reported previously with this model, resistance did not change (10). Consistent with their rapid immune rejection, ^e^allo^S1e^ iECs did not provide any benefit against the development of emphysematous lung disease, and the lung function parameters were similar to *Serpina*^*−/−*^ B6 mice not receiving any cell treatment. In contrast, ^e^alloHIP^S1e^ iEC therapy was effective in preventing emphysematous respiratory patterns as all lung function parameters remained within the range of healthy WT B6 animals. The lungs were then fixed for histology and serially cut for stereological assessment. We found morphometric criteria for emphysema in untreated *Serpina*^*−/−*^ B6 mice, including distal airspace enlargement, loss of alveolar septa, and increase in mean linear intercept (chord) length, a parameter to describe the mean free distance in the air spaces (Fig. 3 *N*–*Q*). Treatment with ^e^alloHIP^S1e^ iECs was successful in preventing all structural damages to the lung morphology. Overall, A1AT replacement via allogeneic cell therapy was able to prevent the development of emphysematous lung disease in *Serpina*^*−/−*^ B6 mice.

### HIP iECs and iCMs for the Treatment of Ischemic Heart Failure.

**Fig. 4.**
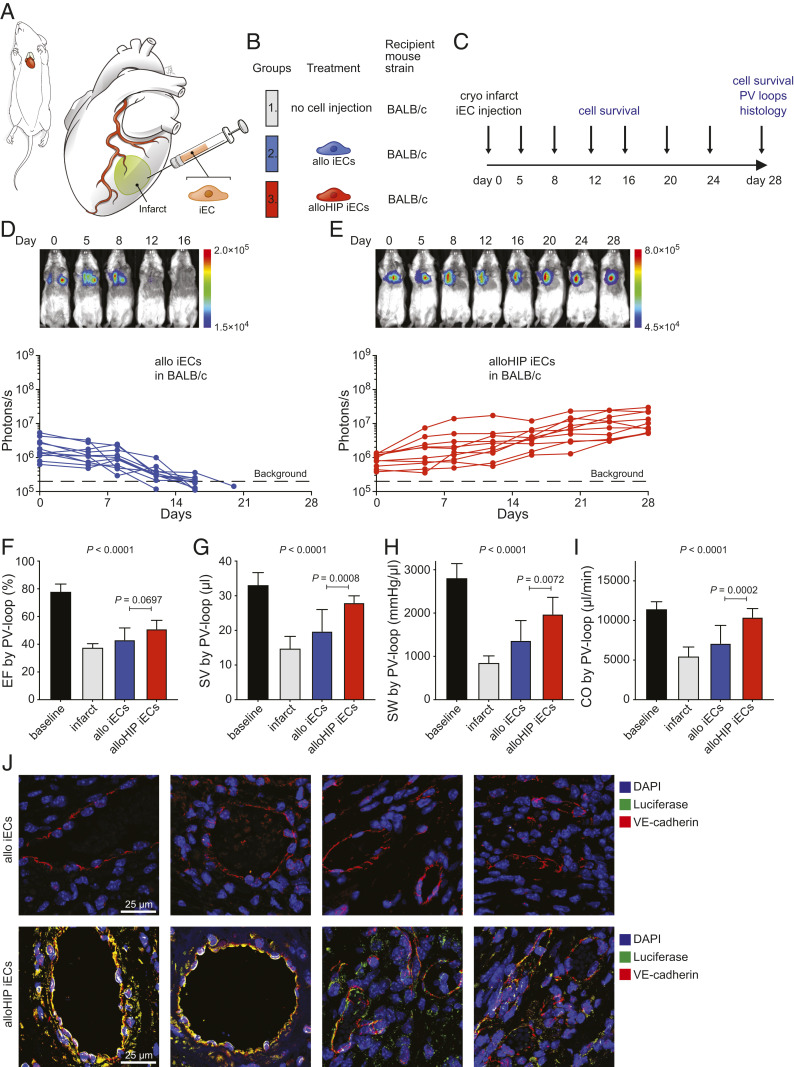
Allogeneic HIP iECs alleviate heart failure in cryoinfarcted hearts. (*A* and *B*) BALB/c mice underwent cryoinfarction of their anterolateral left ventricle (*A*) and some subsequently received injections of allo iECs or alloHIP iECs (*B*) into the border zone. (*C*) Over a study period of 28 d, graft survival was monitored and iEC integration into host myocardium was assessed. (*D* and *E*) The survival of FLuc^+^ allo iEC grafts (*D*, 11 animals) and alloHIP iEC grafts (*E*, 10 animals) was longitudinally followed by BLI. BLI signals of individual animals are plotted, representative pictures are shown. (*F*–*I*) Invasive PV loop analyses (mean ± SD, 10 baseline animals, 4 infarct animals, 10 allo iEC animals, 10 alloHIP animals; ANOVA with Bonferroni post hoc test). Parameters included EF (*F*), SV (*G*), SW (*H*), and CO (*I*). (*J*) Immunofluorescence staining of the infarct border zone did not detect any FLuc^+^ graft cells in recipients of allo iECs. However, all animals that received alloHIP iECs showed engraftment localized to the endothelial cell layers of larger and smaller intramyocardial vessels (representative pictures of 11 animals in the allo and 10 animals in the alloHIP group).

In the first heart experiment, the ability of HIP iECs to treat cryoinjury-induced heart failure was evaluated. BALB/c mice underwent cryoinjury-induced myocardial infarction followed by injections of allo iECs or alloHIP iECs into the infarct border zone (Fig. 4 *A* and *B*) and animals were followed for 28 d (Fig. 4*C*). As expected, allo iECs were rejected within 15 d, while alloHIP iECs survived and proliferated somewhat within the heart (Fig. 4 *D* and *E*). Invasive pressure–volume loop measurements were performed to quantitatively assess hemodynamics during follow-up. The left ventricular ejection fraction markedly dropped with cryoinjury-induced myocardial infarction, and only hearts receiving the alloHIP iECs showed a subtle improvement in contractility over 28 d (Fig. 4*F*). The alloHIP iEC therapy significantly increased left ventricular stroke volume and stroke work, confirming a more physiologic inotropic state of the ventricle (Fig. 4 *G* and *H*). Overall, alloHIP cell therapy led to a significant increase in cardiac output when compared to allo iEC therapy (Fig. 4*I*). The hearts were recovered, processed, and serially cut after 28 d, and immunofluorescence staining showed engrafted FLuc^+^ alloHIP iECs within the injected areas, whereas no FLuc^+^ allo iECs were detected in any hearts (Fig. 4*J*).

**Fig. 5.**
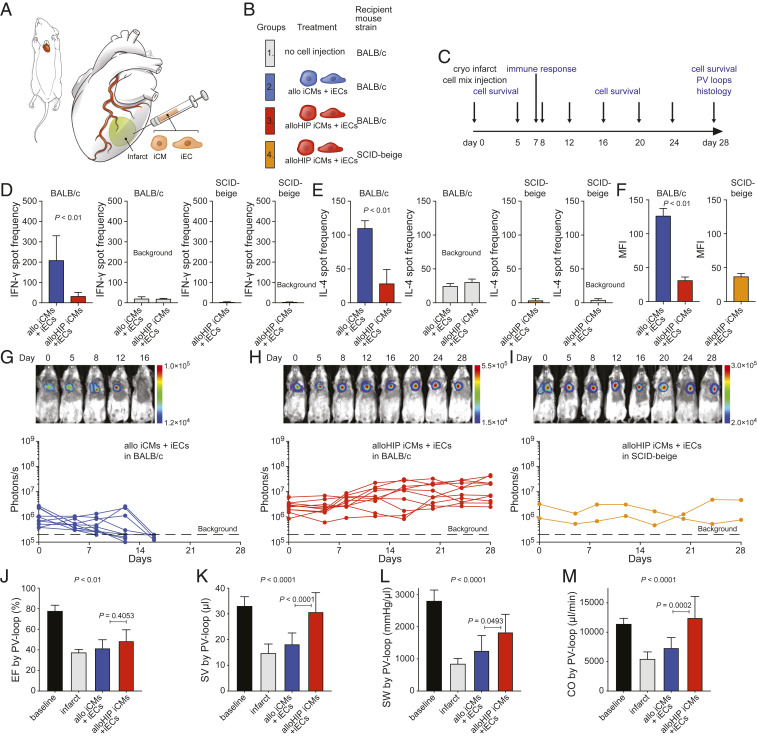
Allogeneic cell mixture of HIP iECs and iCMs alleviates heart failure in infarcted hearts. (*A* and *B*) Mice underwent cryoinfarction of their heart (*A*) and some groups received a mixture of either allo or allHIP iECs and iCMs (*B*). (*C*) The study protocol included early immune assays, longitudinal assessments of cell graft survival, invasive hemodynamic monitoring, and histology after 28 d. (*D*) After 6 d, the donor-specific IFN-γ response of peripheral blood mononuclear cells (PBMCs) was assessed by Elispot assays (mean ± SD, quadruplicates of 11 animals in the allo group and 10 animals in the alloHIP group; two-tailed Student’s *t* test). Background spot frequencies were generated without stimulator cells. SCID-beige animals receiving allHIP iECs and iCMs served as controls (mean ± SD, quadruplicates of 10 animals). (*E*) Simultaneously, the donor-specific IL-4 response of peripheral PBMCs was assessed by Elispot assays (mean ± SD, quadruplicates of 11 animals in the allo group and 10 animals in the alloHIP group; two-tailed Student’s *t* test). Background spot frequencies were generated without stimulator cells. SCID-beige animals receiving alloHIP iECs and iCMs served as controls (mean ± SD, quadruplicates of 10 animals). (*F*) Donor-specific IgM antibodies were assessed on day 6 (mean ± SD, 10 animals per group; two-tailed Student’s *t* test). SCID-beige animals receiving alloHIP iECs and iCMs served as controls (mean ± SD, 5 animals). (*G*–*I*) The survival of FLuc^+^ allo (*G*, 10 animals) and alloHIP iEC and iCM grafts (*H*, 9 animals) in allogeneic BALB/c mice was longitudinally followed by BLI. All alloHIP cell grafts also survived in SCID-beige mice (*I*, 2 animals). BLI signals of individual animals are plotted, representative pictures are shown. (*J*–*M*) Invasive PV loop analyses (mean ± SD, 10 baseline animals, 4 infarct animals, 9 allo animals, 10 alloHIP animals; ANOVA with Bonferroni post hoc test). Parameters included EF (*J*), SV (*K*), SW (*L*), and CO (*M*).

Next, a mixture of allo or alloHIP iECs and iCMs was used to improve remuscularization (Fig. 5 *A*–*C*). A more thorough assessment of the recipient immune response was conducted in this study. After 7 d, there was a vigorous immune cell and antibody response against the allo iEC and iCM grafts (Fig. 5 *D*–*F*) but no measurable immune response against the alloHIP cell mixture. All allo cell grafts were rejected within 14 d, but all alloHIP grafts survived and proliferated in the hearts of allogeneic BALB/c mice (Fig. 5 *G* and *H*). The survival of the alloHIP cell mixture in allogeneic recipients was not even inferior to their survival in severely immunodeficient SCID-beige mice, further supporting the absence of any relevant allo-immune responses in BALB/c (Fig. 5*I*). Pressure–volume loop measurements again showed a nonsignificant improvement in the ejection fraction (EF) over 28 d (Fig. 5*J*). The alloHIP cell mixture significantly increased left ventricular stroke volume (SV), stroke work (SW), and cardiac output (CO) when compared to allo cell therapy (Fig. 5 *K*–*M*). There was no difference between the hemodynamic parameters after alloHIP iEC therapy and alloHIP iEC and iCM therapy. The hearts were then cut into serial sections (*SI Appendix*, Fig. S6) and underwent histologic assessment. The cryoinjury resulted in very reproducible infarcts consisting of mostly fibrous replacement of the anterior and lateral left ventricular (LV) free wall, from the anterior septum to the posteromedial papillary muscle. There were a few chronic inflammatory cells, which appeared to be macrophages and lymphocytes, mostly at the edges of the fibrosis and without notable differences between groups. This response appeared typical for a reparative postinfarct inflammation. No undifferentiated cells, teratomas, or tumor formation were seen in any of the samples. In the alloHIP group, hearts had a thicker interventricular septum and posterior LV with a lack of dilatation. We could detect engraftment of transplanted FLuc^+^ cells in the alloHIP-treated hearts (*SI Appendix*, Fig. S7). We could not detect any survival of allo graft cells in any of the hearts. Together our data show that alloHIP cell therapy was able to improve hemodynamics in allogeneic mice with myocardial infarction.

## Discussion

This study shows that HIP-derived cell therapeutics can successfully be employed in regenerative medicine to treat diseases for which there are no good treatment options available. We chose three major diseases affecting different organ systems to explore the versatility of HIP cell therapy. All three models, however, have sudden injury triggers, and a critical disease progression happens expeditiously after the initial event. This allows for standardized modeling of diseases in mice, although the progression in humans may be more chronic and protracted. It also allows for standardized assessment and comparison of the allo and alloHIP treatment groups in a convenient time period because any therapeutic effect will have to mainly mitigate the initial tissue injury in order to achieve a benefit. To underline the translational spirit of these studies, we aimed for subjective and clinically relevant endpoint measures used in clinical human trials.

Peripheral arterial disease has a prevalence of approximately 10% in individuals ≥40 y of age (12), and annually 11.2% of patients with peripheral arterial disease develop CLI, defined as chronic ischemic rest pain, ulcers, or gangrene (13). Clinically, CLI is associated with poor outcomes, since 30% of cases require amputation and 25% of patients will die within 1 y. Half of all patients with CLI are not candidates for surgical or percutaneous revascularization (14) and alternative cell-based therapies are desperately needed. So far, cell therapy trials have shown promising efficacy in patients with CLI when using peripheral blood or bone marrow mononuclear cells or more narrowly defined CD34^+^ or CD133^+^ stem cells (15). Clinically, the most relevant primary outcome endpoint used in randomized controlled trials is the amputation rate. Our data show that alloHIP iEC therapy markedly increased limb preservation in an established mouse model for CLI. We could further correlate limb preservation with improved limb perfusion, a common secondary endpoint in clinical trials (16). Laser Doppler imaging is a very useful tool to noninvasively assess global blood perfusion in the mouse hindlimb without the use of tracer dyes (8, 17). BLI studies showed survival of transplanted alloHIP iECs in allogeneic recipients and histologically, we observed engraftment. FLuc^+^ graft cells mainly localized along the endothelial layer of vascular structures, although we could not differentiate between the integration into existing host architecture and neovascularization. Prior animal studies showed that neovascularization occurs with transplanted endothelial progenitor cells (8). Clinical trials using cell therapy for CLI have so far only used autologous, patient-derived cells (15), which to a large extent have not even been well characterized or dedicated to the endothelial lineage. The prospect of developing universal, allogeneic off-the-shelf iEC therapeutics could advance cost-effective and widespread options to treat CLI.

Chronic obstructive pulmonary disease is the third leading cause of death worldwide (18), and it affects about 10% of the world population (19). Besides environmental risk factors, A1AT deficiency is the most common genetic mutation (20) located in the *SERPINA1* gene. Insufficient levels of the serum protease inhibitor A1AT (21) cause an imbalance in the alveolar interstitium with unopposed NE activity leading to a destruction of the alveolar walls and lung parenchyma. Patients with lung disease from A1AT deficiency are currently being treated with standard medical therapy (22), intravenous augmentation therapy, or require lung transplantation for respiratory failure (23). Although the infusion of pooled human A1AT is currently most efficient to elevate A1AT in the plasma and lung interstitium (24), it is very expensive and has not convincingly improved clinical endpoints in trials (25, 26). Specific mouse models for A1AT deficiency have long been missing and instillation of porcine pancreatic elastase or human NE have been used to create the protease/antiprotease imbalance (27). The quintuple *Serpina1a–e* knockout B6 mouse used herein is the first animal model of A1AT deficiency that allows precise assessments of disease mechanisms linked to the underlying genetic mutations. Commonly used endpoints in clinical trials include measurements of lung function and lung density. We used various FlexiVent maneuvers to assess lung function in mice and were able to identify emphysematous disease in untreated *Serpina*^*−/−*^ B6 mice. Transplantation of ^e^alloHIP^S1e^ iECs did not only normalize serum A1AT levels but also prevented deterioration of lung function. We used histology to assess lung structure and could correlate the preserved lung function in the treatment group with preserved alveolar septal tissue architecture. Our studies show that ectopic A1AT production by alloHIP iECs can restore plasma levels and prevent emphysema development.

Heart failure (HF) is a rising global epidemic (28) with more than 5.7 million patients in the United States, 870,000 new cases every year (29), and an increase in hospitalizations for HF (30). Clinical trials on cell therapy for heart failure have been conducted for almost two decades, so far with disappointing overall results (31). Objective clinical endpoints may include survival, number of events or hospitalizations, and subjective endpoints like symptom score and health-related quality of life (32, 33), but are difficult to use in mouse models. Instead, surrogate efficacy endpoints that correlate with clinical endpoints like hemodynamic improvement have been proposed (34). Invasive hemodynamic testing is best suited to assess cardiac output in mice and currently the gold standard tool (35–37). Our studies showed a significant treatment benefit for alloHIP iECs with improved hemodynamics. We observed survival and engraftment of alloHIP iECs in the areas of injection into ischemic myocardium. ECs promote cardiomyocyte survival and spatial reorganization via paracrine signaling (38), possibly involving the release of cytokines (39) or exosomes (40). While beneficial effects of endothelial progenitor cells have been described in immunodeficient mouse models (41) and in clinical trials using autologous cells (42), we herein show the use of universal cell products in immunocompetent allogeneic recipients.

In order to support cardiac remuscularization, we then added equal amounts of CMs to the EC injections and again observed improved hemodynamic parameters with alloHIP cells as compared to allo cells. The alloHIP cell grafts survived in allogeneic recipients and engrafted in the cardiac wall. A recent study indicated the contribution of transplanted cardiomyocytes to improved vascularity in a preclinical model of myocardial infarction as a mechanism to enhance cardiac function (43). There was no obvious added benefit to hemodynamic recovery by the cotransplanted alloHIP CMs in our study, maybe because the cell amounts used were not sufficient to achieve relevant remuscularization. Alternatively, there might not be a linear correlation between cell amount and cardiac recovery as hinted by the similar results achieved across a wide range of cell doses reported by other groups (44, 45). Beneficial effects from transplantation of xenogeneic (43, 46, 47) or allogeneic (48, 49) iCMs had so far only been shown in animals treated with heavy immunosuppression. Here we report success with allogeneic cells in immunocompetent mice and show no measurable immune activation. Large-scale manufacturing of iCMs has been developed (50) and cell delivery to the heart is established (51). With the immunological barrier for immunosuppression-free allogeneic cell transplantation resolved, iCM engraftment issues, including avoidance of arrhythmogenic complications as described in pig and nonhuman primate studies, need to be overcome (47, 48).

Since the first description of HIP cells, many different derivatives and implant sites have been tested, and we have not yet encountered an allogeneic immune response. Universal off-the-shelf cell products can be a major factor in preventing spiraling of costs for regenerative medicine as has been encountered with novel cancer therapeutics (52). This study therefore provides a realistic outlook for advancements of affordable allogeneic cell therapies for large patient populations.

## Materials and Methods

### Mice.

BALB/c (BALB/cJ, H2^d^), C57BL/6 (C57BL/6J, H2^b^), and SCID-beige (CBySmn.CB17-Prkdcscid/J) (all 6 to 12 wk) were purchased from The Jackson Laboratory. *Serpina*^*−/−*^ C57BL/6 mice were generated by C.M. as reported previously (10). The number of animals per experimental group is presented in each figure. Mice received humane care in compliance with the Guide for the Principles of Laboratory Animals. Animal experiments were approved by the University of California, San Francisco (UCSF) Institutional Animal Care and Use Committee and performed according to local guidelines.

### Mouse iPSC culture.

Mouse C57BL/6 iPSCs were generated from tail tip fibroblasts as reported previously (3). Briefly, iPSCs were grown in iPSC medium on confluent mouse embryonic fibroblast (MEF) feeder cells. Medium was changed daily, and cells were passaged every 2 to 3 d. Mouse iPSCs were cultured on gelatin (Millipore) without feeders prior to experiments. Cell cultures were regularly screened for mycoplasma infections using the MycoAlert Kit (Lonza). Gene editing was performed as described previously (3).

### Transduction to Express Firefly Luciferase.

A total of 100,000 iPSCs were plated in gelatin-coated six-well plates and incubated overnight at 37 °C at 5% CO_2_. Media were changed the next morning and 200 μL of FLuc lentiviral particles (10^7^ IFU/mL, GenTarget) was added. After 36 h, 1 mL of cell media was added and the next day, a complete media change was performed. After another 2 d, luciferase expression was confirmed by adding D-luciferin (Promega). Signals were quantified with the Ami HT system (Spectral Instruments Imaging) in maximum photons s^−1^ cm^−2^ per steradian.

### Derivation and Characterization of iECs.

Mouse iPSCs were plated on gelatin in 6-well plates and maintained in iPSC media until they reached 60% confluency. Differentiation into iECs was performed as described in detail previously (3). Cells after differentiation underwent magnetic-activated cell sorting (MACS) purification using negative selection with anti-CD15 mAb-coated magnetic microbeads (Miltenyi). The iEC phenotype was confirmed by immunofluorescence (IF) for expression of Cd31 (ab28364, Abcam), and VE-cadherin (sc-6458, Santa Cruz Biotechnology) with secondary antibodies conjugated with AF488 or AF555 (Invitrogen). For the tube formation assay, 2.5 × 10^5^ iECs were stained with 5 µM carboxyfluorescein succinimidyl ester (CFSE) and 0.1 μg/mL Hoechst (both Thermo Fisher) for 10 min at room temperature and plated on 10 mg/mL undiluted Matrigel (356231, Corning) in 24-well plates. After 48 h, tube formations were visualized by IF. PCR was performed using primers VE-cadherin (Cdh5): (forward) 5′-GGA​TGC​AGA​GGC​TCA​CAG​AG-3′, (reverse) 5′-CTG​GCG​GTT​CAC​GTT​GGA​CT-3′. All other primers were included in the Mouse ES/iPS Cell Pluripotency RT-PCR Kit (ASK-6001, Applied StemCell).

### Gene Editing to Generate A1AT-Secreting iECs.

The *Serpina1e* (NM_009247.2) cDNA was synthesized and cloned into a lentivirus with zeocin resistance (Thermo Fisher Scientific), which was used to transduce B6 and B6HIP iECs followed by antibiotic selection and expansion of B6^S1e^ and B6HIP^S1e^ iECs. The cells were then transduced to express allogeneic major (H-2K^d^) and minor (BALB/c variant of Co3) histocompatibility antigens using lentiviral particles (both Gentarget). These MHC-engineered ^e^allo^S1e^ and ^e^alloHIP^S1e^ iECs were thus allogeneic to the *Serpina*^*−/−*^ C57BL/6 recipient mice.

### Derivation and Characterization of iCMs.

Differentiation into iCMs was performed as described in detail previously (3). Beating cells developed around days 11 to 14. Cells then underwent MACS purification using negative selection with anti-CD15 mAb-coated magnetic microbeads (Miltenyi). IF staining was performed using primary antibodies against alpha-sarcomeric actinin (EA-53, Abcam) and Troponin I (ab47003, Abcam) followed by the corresponding secondary antibodies conjugated with AF488 or AF555 (Invitrogen). The following primers were Gata4: (forward) 5′-CTG​TCA​TCT​CAC​TAT​GGG​CA-3′, (reverse) 5′-CCA​AGT​CCG​AGC​AGG​AAT​TT-3′; Myh6: (forward) 5′-ATC​ATT​CCC​AAC-​GAG​CGA​AAG-3′, (reverse) 5′-AAG​TCC​CCA​TAG​AGA​ATG​CGG-3′. All other primers were included in the Mouse ES/iPS Cell Pluripotency RT-PCR Kit (ASK-6001, Applied StemCell).

### Flow Cytometry.

Mouse iECs were labeled with a goat anti-mouse VE-cadherin primary antibody (sc-6458, Santa Cruz Biotechnology) and AF488 (A11055, Invitrogen) or AF555 (A21432, Invitrogen) conjugated donkey anti-goat IgG secondary antibodies (both Invitrogen). Mouse iCMs were treated with Fix/Perm solution (BD Bioscience) for 20 min at room temperature. The cells were subsequently washed twice and then labeled with fluorescein isothiocyanate (FITC)-conjugated anti-cardiac troponin T antibody (130-119-575, clone REA400, Miltenyi) or FITC-conjugated recombinant human IgG1 isotype control antibody (130-118-354, clone: REA293, Miltenyi). The expression of VE-cadherin and troponin T was assessed by flow cytometry (FACS Aria Fusion or FACS Calibur, BD Bioscience) and the analysis was performed using FlowJo software.

### Elispot assays.

For unidirectional Enzyme-Linked ImmunoSpot (Elispot) assays, recipient splenocytes were isolated from spleen 6 d after cell injection and used as responder cells (3). Donor cells were mitomycin-treated (50 μg/mL for 30 min) and used as stimulator cells. A total of 100,000 stimulator cells were incubated with 1 × 10^6^ recipient responder splenocytes for 24 h and IFN-γ and IL-4 spot frequencies were enumerated using an Elispot plate reader (AID GmbH).

### Donor-Specific Antibodies.

Sera from recipient mice were decomplemented by heating to 56 °C for 30 min as described previously (3). Equal amounts of sera and cell suspensions (5 × 10^6^ /mL) were incubated for 45 min at 4 °C. Cells were labeled with FITC-conjugated goat anti-mouse IgM (Sigma-Aldrich) and analyzed by flow cytometry (FACS Calibur, BD Bioscience).

### Graft Survival by BLI.

D-luciferin firefly potassium salt (375 mg/kg; Biosynth AG) was dissolved in phosphate-buffered saline (PBS) (pH 7.4, Gibco, Invitrogen) and injected intraperitoneally (i.p.) (250 μL per mouse) into anesthetized mice. Animals were imaged using the Ami HT system (Spectral Instruments Imaging). Region of interest (ROI) bioluminescence was quantified in units of maximum photons s^−1^ cm^−2^ per steradian. The maximum signal from a ROI was measured using Aura Image software (Spectral Instruments Imaging).

### Histology.

Tissue was recovered and fixed in 4% paraformaldehyde in PBS for 24 h. Samples were dehydrated, embedded in paraffin, and cut into sections of 5 µm thickness. For immunofluorescence, sections were rehydrated and underwent antigen retrieval and blocking. Samples were incubated with antibodies against luciferase (ab21176), VE-cadherin (sc-6458), or alpha-sarcomeric actinin (EA-53, Abcam) and a corresponding secondary antibody was conjugated with AF488 or AF555 (Invitrogen). Cell nuclei were counterstained with DAPI and images taken with a Leica SP5 laser confocal microscope (Leica).

### Mouse Hindlimb Ischemia Model.

BALB/c mice were anesthetized with isoflurane. One million iECs were resuspended in 100 μL saline and mixed with 100 μL Matrigel (Corning, 356231) prior to the injection. The femoral artery was exposed through a 2-cm skin incision. The femoral artery was ligated with 6-0 prolene (Ethicon) and excised from its proximal origin as a branch of the external iliac artery to the distal point where it bifurcates. Eight injections with 25 μL cell suspension were made with a Hamilton 22-G syringe, intramuscularly around the removed artery. A laser Doppler perfusion imager (moorLDI2-IR, Moor Instruments) was used to sequentially measure the blood flow in the hindlimbs over time. Digital color-coded images were analyzed to quantify the blood flow in the region from the knee joint to the toe, and the mean perfusion units were calculated.

### Mouse Lung Emphysema Model.

WT or *Serpina*^*−/−*^ C57BL/6 mice received two sequential orotracheal doses of LPS to induce lung disease with 1 μg in 30 μL saline for the first dose and 0.5 μg in 30 μL for the second dose (serotype 055:B5 *Escherichia coli* LPS, L2880; Sigma-Aldrich). Mice were anesthetized with an i.p. dose of a ketamine/xylazine mixture (90 mg/kg of ketamine and 4.5 mg/kg of xylazine) and placed in dorsal recumbency on a rodent work stand (Braintree Scientific) and intubated. Following instillation, mice received three ventilations with 0.2 mL of air.

### Quantification of A1AT.

The mouse A1AT ELISA Kit (ab205088, Abcam) was used according to the manufacture’s protocol. Briefly, serum samples or cell culture supernatant were incubated with an AAT antibody, followed by incubation with an horseradish peroxidase (HRP)-conjugated secondary antibody and a peroxidase substrate. A microplate reader with an absorbance of OD450 nm (Molecular Devices) was used to measure the AAT level of the standards and study samples.

### FlexiVent.

Mice were anesthetized as outlined above. A tracheotomy was performed, and a precalibrated cannula was introduced into the trachea. The mouse was then placed on a computer-controlled piston-ventilator FlexiVent system (Scireq) and ventilated at a tidal volume of 10 mL/kg, at a rate of 150 breaths per minute, and a positive end expiratory pressure of 3 mmHg. Neuromuscular blockade with pancuronium bromide (2.5 mg/kg) was given to prevent spontaneous respiratory effort. Measurements were obtained as previously described (10).

### Lung Stereology.

Immediately following measurements of lung mechanics (FlexiVent), lungs were harvested for stereological analysis. The chest was opened and the pulmonary circulation flushed via right ventricular puncture with 10 mL cold PBS. The degassed lung was inflated with 1% ultra-low temperature gel agarose (Sigma-Aldrich) in neutral buffered paraformaldehyde (4%) to 25 cm pressure. Subsequently, lung tissues were oriented randomly in cassettes and embedded in paraffin, followed by sectioning started at random depth at a uniform thickness (5 μm). Slides were stained with hematoxylin and eosin and 10 randomly oriented nonoverlapping fields from each section were photographed. Lung morphology was quantified using Stepanizer software (https://www.stepanizer.com/). Surrogate markers for lung morphology were calculated as follows (53, 54): Volume fraction of alveolar septal tissue: *Vv*(*sept*/*par*) = ∑ *P*(*sept*)/ ∑ *P*(*par*), fraction of alveolar volume: *Vv*(*alv*/*par*) = ∑ *P*(*alv*)/ ∑ *P*(*par*), surface density of alveolar septa: *Sv*(*sept*/*par*) = (2 × ∑ *I*)/(∑ *P*(*par*) × *l*/*p*), mean linear intercept: Lm = 2⋅k⋅d⋅P(asp)/I(A).

### Mouse Myocardial Cryoinfarction Model.

A recently developed cryoinjury model was used (55). Briefly, after induction of analgesia and anesthesia, the mice were intubated and a right thoracotomy was performed. A mini-Goldstein retractor (Fine Science Tools) was used to spread the ribs. With the blunt forceps, the pericardium was opened, and the heart was exposed. Cryoinfarction was produced by applying a cryoprobe of 3 mm in diameter (Cry-AC-3 B-800, Brymill Cryogenic Systems) to the anterolateral LV free wall followed by freezing for 10 s. The position of the probe was carefully chosen using the left anterior descending artery, the left atrium, and pulmonary artery as anatomic landmarks. Rinsing with saline at room temperature allowed nontraumatic detachment of the probe from the LV wall after the freezing. A total of 500,000 iECs and 500,000 iCMs were resuspended in 20 μL saline, and 5 μL Matrigel (356231, Corning) was added to the cells prior to the injection. Two injections were made with a Hamilton 30-G syringe both anterior and lateral to the infarction area.

### Pressure–Volume Loop (PV Loop).

PV loops of the LV were acquired and analyzed as previously described (56). Briefly, hemodynamic measurements were performed using a 1.2 Fr PV conductance catheter with the ADV500 PV measurement system (Transonic). Mice received buprenorphine (0.1 mg/kg) subcutaneously (s.c.) before initiating 5% (vol) isoflurane inhalation and were then mechanically ventilated and maintained at 0.5 to 1% (vol) isoflurane during the surgical procedure. An incision was performed above the xyphoid process, until the diaphragm became clearly visible from beneath. After cutting through the diaphragm to expose the heart, a stab wound near the apex was made using a 27-G needle. The PV catheter tip was inserted into the LV. After stabilization of the signal for 10 min, baseline PV loops at steady state or at varying preloads during the inferior vena cava occlusions were recorded. LabChart v.8 software (AdInstruments) was used for data analysis.

### Statistics.

All data are expressed as mean ± SD or in box plot graphs showing the median and the minimum to maximum range. Intergroup differences were appropriately assessed by either unpaired Student’s *t* test or one-way analysis of variance (ANOVA) with Bonferroni post hoc test.

## Supplementary Material

Supplementary File

## Data Availability

All study data are included in the article and/or *SI Appendix*.
