# Supplementary material for: Hypoimmune induced pluripotent stem cell–derived cell therapeutics treat cardiovascular and pulmonary diseases in immunocompetent allogeneic mice
Source: Proc Natl Acad Sci U S A. 2021 Jul 9;118(28):e2022091118. doi: 10.1073/pnas.2022091118 (PMC8285900; doi:10.1073/pnas.2022091118)
Supplement: Supplementary File [file pnas.2022091118.sapp.pdf]

PNAS
www.pnas.org

Supplementary Information for

Hypoimmune iPSC-derived cell products treat cardiovascular and pulmonary diseases in immunocompetent allogeneic mice

Tobias Deuse[1*], Grigol Tediashvili[1,2*], Xiaomeng Hu[1,2,3,4*], Alessia Gravina[1], Annika Tamenang[1,2], Dong Wang[1], Andrew Connolly[5], Christian Mueller[6,7], Beñat Mallavia[8], Mark R. Looney[8,9], Malik Alawi[10], Lewis L. Lanier[11#], Sonja Schrepfer[1,4#]

[1] Department of Surgery, Division of Cardiothoracic Surgery, Transplant and Stem Cell Immunobiology (TSI)-Lab, University of California San Francisco, San Francisco, California, USA;
[2] Department of Cardiovascular Surgery, University Heart Center Hamburg, Hamburg, Germany;
[3] German Center for Cardiovascular Research (DZHK), partner site Hamburg/Kiel/Luebeck, Hamburg, Germany;
[4] Sana Biotechnology Inc., 1 Tower Place, South San Francisco, CA, USA.
[5] Department of Pathology, University of California-San Francisco, San Francisco, California, USA.
[6] Horae Gene Therapy Center, University of Massachusetts, Worcester, Massachusetts, USA;
[7] Department of Pediatrics, University of Massachusetts, Worcester, Massachusetts, USA;
[8] Department of Medicine, University of California San Francisco, San Francisco, California, USA;
[9] Department of Laboratory Medicine, University of California San Francisco, San Francisco, California, USA;
[10] Bioinformatics Core, University Medical Center Hamburg-Eppendorf, Hamburg, Germany;
[11] Department of Microbiology and Immunology and the Parker Institute for Cancer Immunotherapy, University of California San Francisco, San Francisco, California, USA.

*Authors share first authorship
#Authors share senior authorship

Corresponding author: Sonja Schrepfer

Email: Sonja.Schrepfer@ucsf.edu

This PDF file includes:

Figures S1 to S7

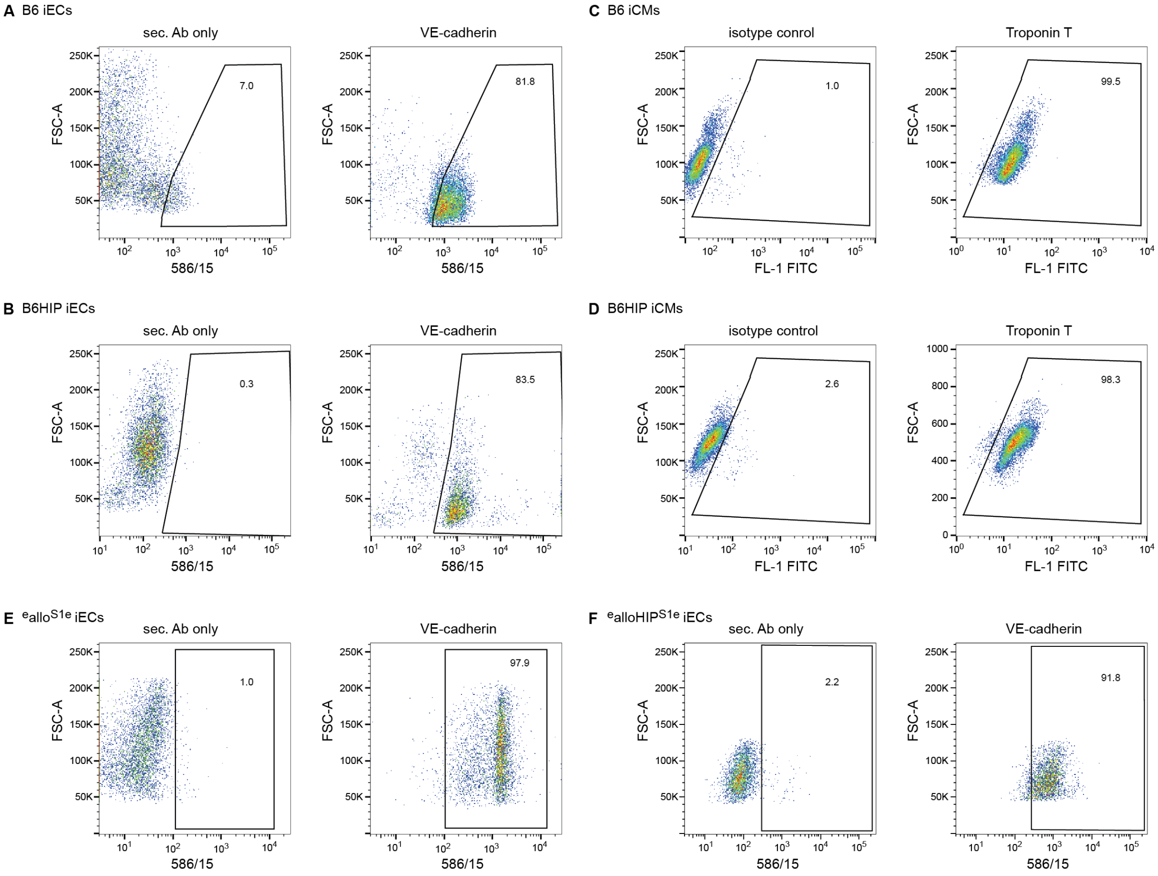

Fig. S1: *Purity of iECs and iCMs.* (*A-F*) B6 iECs (A) or B6HIP iECs (B), ^{e}allo^{S1e} iECs (E) or ^{e}alloHIP^{S1e} iECs (F) underwent quality control by flow cytometry. Successfully differentiated iECs were defined as being VE-cadherin+ (representative scatter blots from 3 independent experiments). Similarly, B6 iCMs (C) or B6HIP (D) iCMs were tested for their troponin T expression (representative scatter blots from 3 independent experiments).

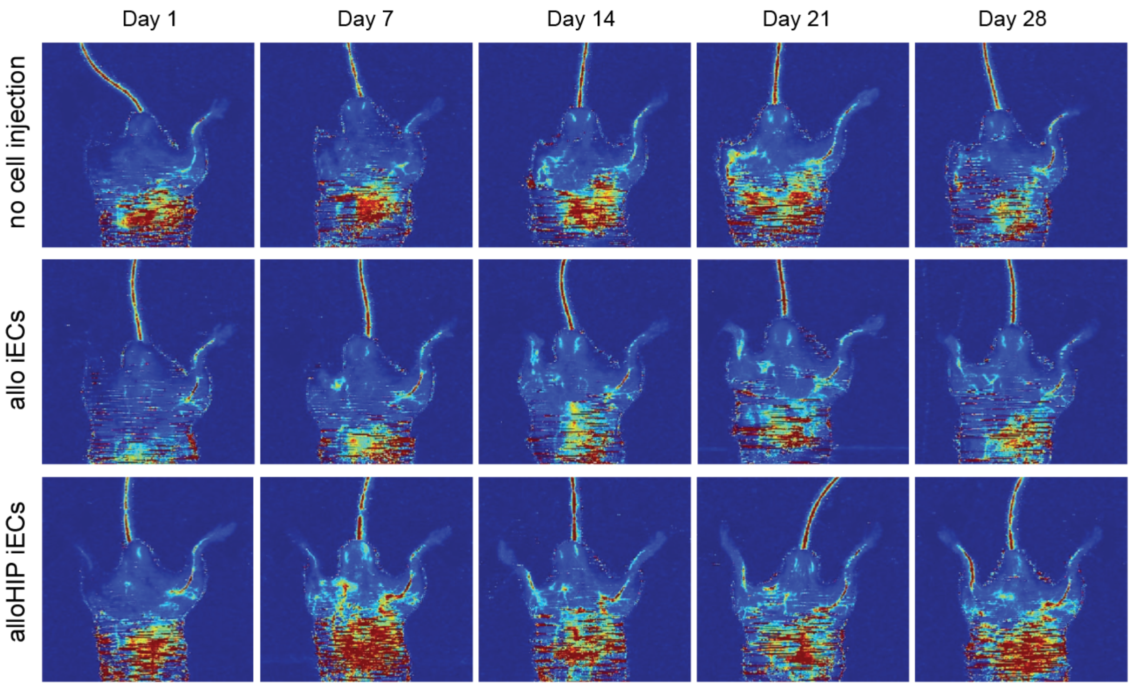

Fig. S2: *Hindlimb doppler.* Limb perfusion in mice with CLI was sequentially assessed by laser Doppler imaging. Five time points were picked after ligation and excision of the left proximal superficial femoral artery (representative pictures of 15 animals with no cell injection, 5 animals in the allo iEC group and 15 animals in the alloHIP iEC group).

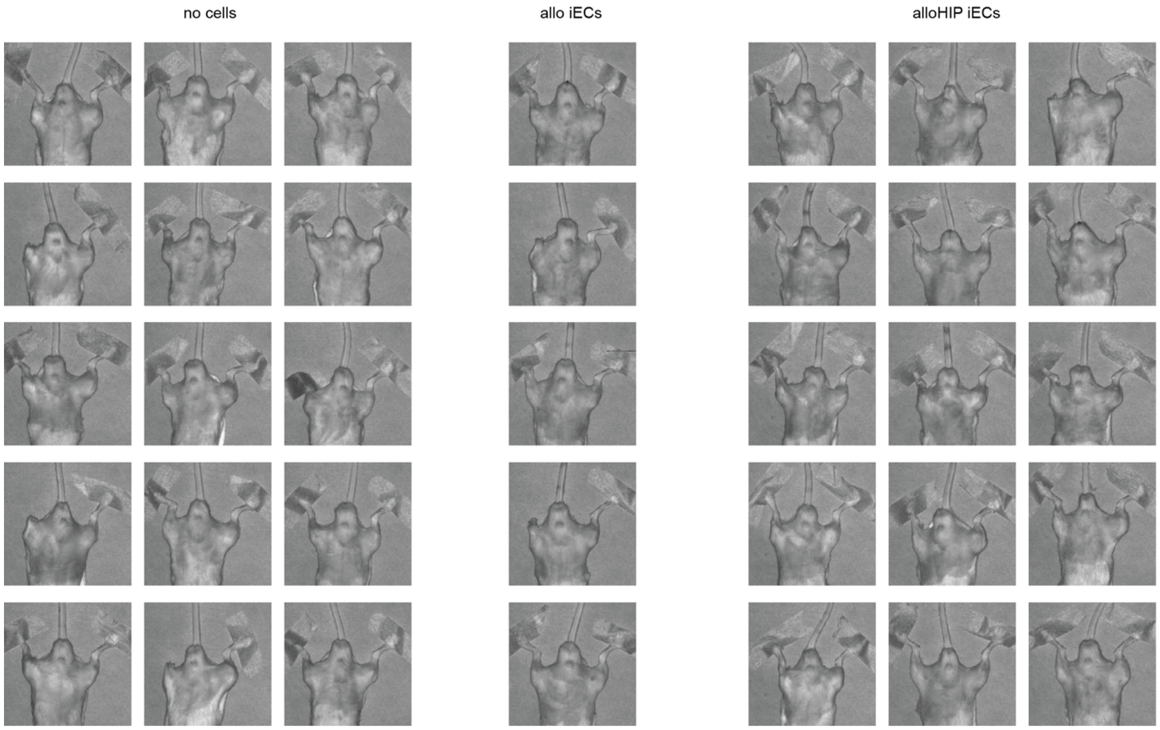

Fig. S3: *Hindlimb status.* After 28 days, the ischemic hindlimbs were assessed and photographed. All animals are shown.

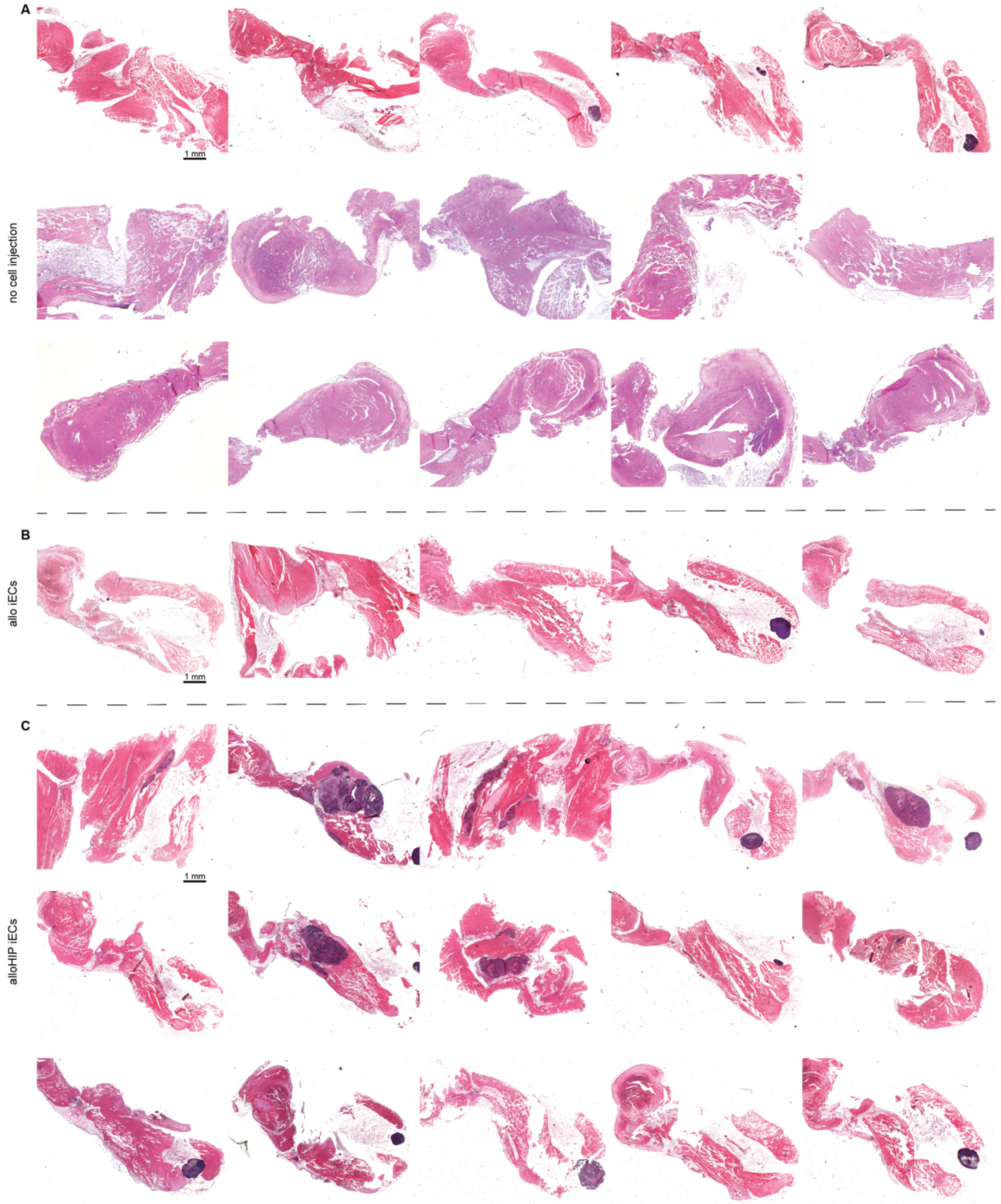

Fig. S4: *Hindlimb histology.* (*A-C*) The groin and thigh areas around the resected femoral artery, which contain the cell injections, were recovered, serially cut and stained with hematoxylin and eosin. Sections through the middle of this area at similar anatomic levels are shown for all animals in the no cell injection group (A), the allo iEC group (B) and the alloHIP iEC group (C).

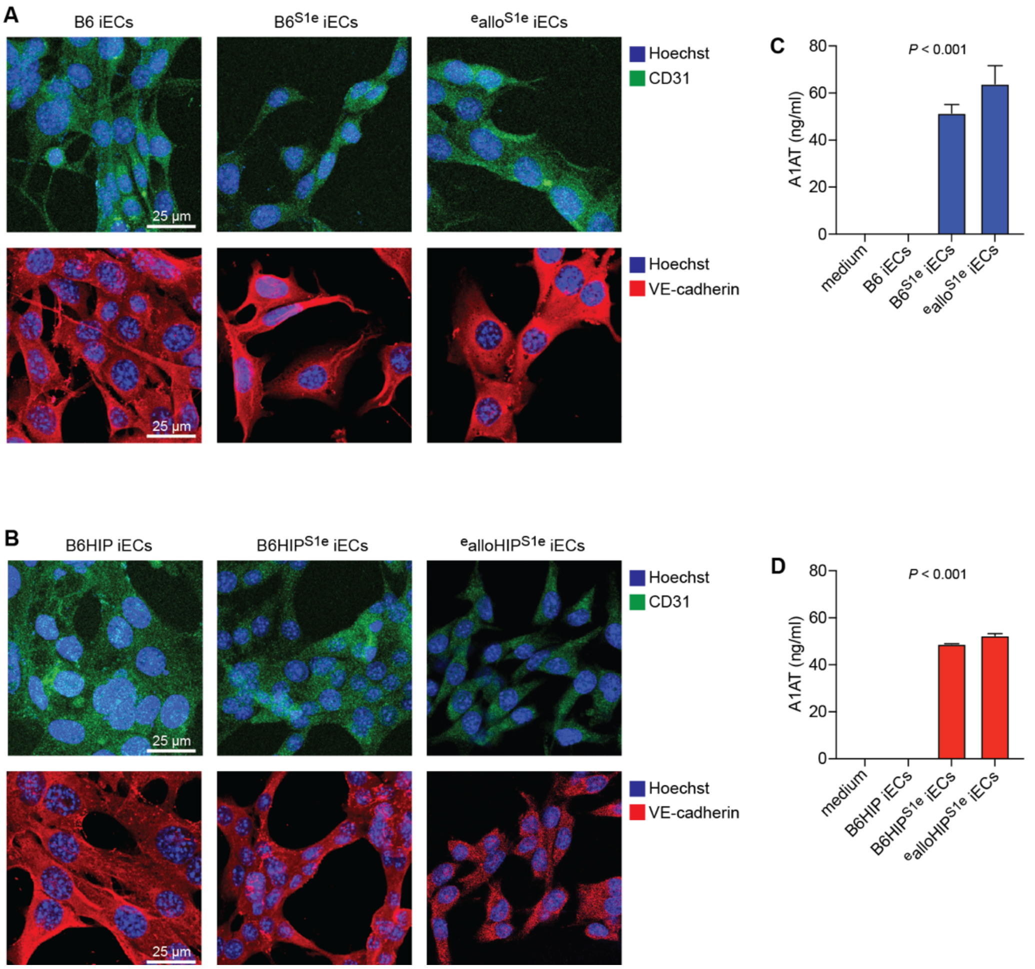

Fig. S5: *A1AT-releasing $^{e}allo^{S1e}$ and $^{e}alloHIP^{S1e}$ iECs*. (*A* and *B*) B6, B6^{S1e}, and ^{e}allo^{S1e} iECs (A) or B6HIP, B6HIP^{S1e}, and ^{e}alloHIP^{S1e} iECs (B) were grown in culture and shared typical endothelial cell features (representative pictures of 2 independent experiments). (*C* and *D*) A total of 1.5 × 10^{5} B6, B6^{S1e}, and ^{e}allo^{S1e} iECs (C) or B6HIP, B6HIP^{S1e}, and ^{e}alloHIP^{S1e} iECs (D) iECs were cultured for 24 h in medium. The A1AT concentration was then assessed by Elisa (mean ± s.d., 3 independent experiments, ANOVA).

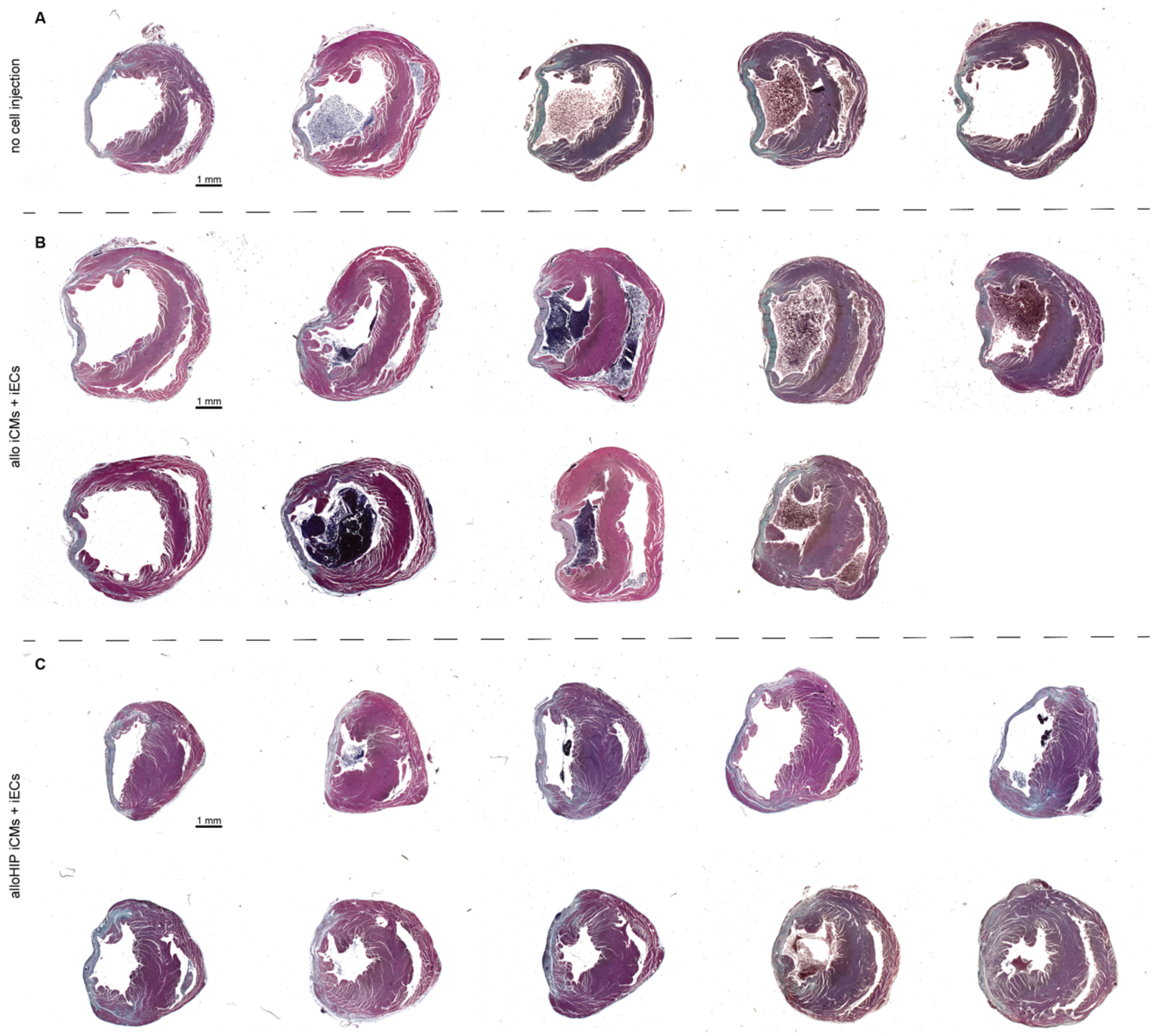

Fig. S6: *Histological evaluation of hearts.* (*A*-*C*) Explanted hearts were serially sectioned and stained with Masson's Trichrome. Sections through the mid cavity are shown for all animals in the no cell injection group (A), the allo iCM + iEC group (B) and the alloHIP iCM + iEC group (C).

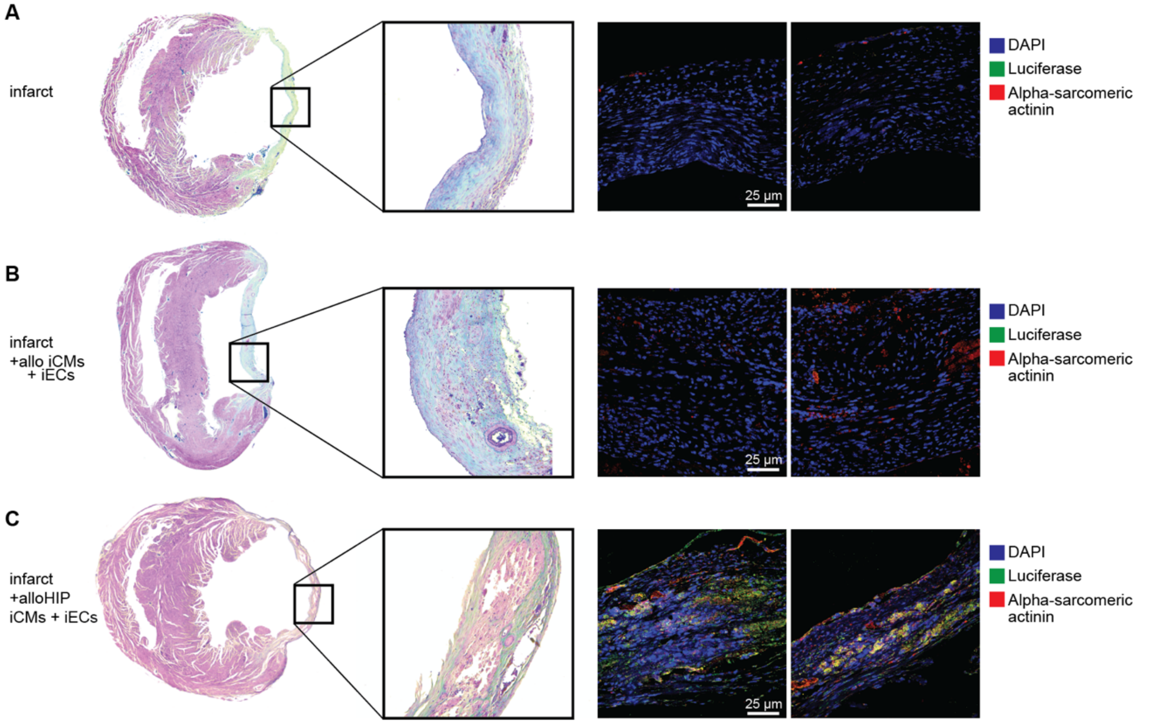

Fig. S7: *Histological evaluation of iCM engraftment.* (*A*-*C*) Sections of cryoinfarcted hearts without cell therapy (A) and infarcted heart that were injected with allo (B) or alloHIP iECs and iCMs (C) were stained for immunofluorescence. The scar areas were screened for transplanted cells. No $FLuc^+$ cells were found in hearts without cell injections or with injections of the allo cell mixture. In hearts injected with alloHIP cell mixture, we found several islands that stained positive for FLuc and alpha-sarcomeric actinin, suggesting engrafted alloHIP iCMs.
